# Dynamic Changes in Fermentation Quality and Structure and Function of the Microbiome during Mixed Silage of *Sesbania cannabina* and Sweet Sorghum Grown on Saline-Alkaline Land

**DOI:** 10.1128/spectrum.02483-22

**Published:** 2022-10-03

**Authors:** Tianwei Wang, Jiaqi Zhang, Weixiong Shi, Jiahao Sun, Tianqi Xia, Fuqing Huang, Yayong Liu, Huangkeyi Li, Kunling Teng, Jin Zhong

**Affiliations:** a State Key Laboratory of Microbial Resources, Institute of Microbiology, Chinese Academy of Sciences, Beijing, China; b School of Life Sciences, University of Chinese Academy of Sciences, Beijing, China; c School of Life Sciences, Yunnan University, Kunming, China; State Key Laboratory of Microbial Resources, Institute of Microbiology, Chinese Academy of Sciences

**Keywords:** *Sesbania cannabina*, sweet sorghum, mixed-silage quality, bacterial community, microbial function prediction

## Abstract

Protein-rich Sesbania cannabina and sugar-rich sweet sorghum [Sorghum dochna (Forssk.) Snowden] are characterized by their higher tolerance to saline-alkaline stresses and simultaneous harvests. They could be utilized for coensiling because of their nutritional advantages, which are crucial to compensate protein-rich forage in saline-alkaline regions. The current study investigated the fermentation quality, microbial community succession, and predicted microbial functions of *Sesbania cannabina* and sweet sorghum in mixed silage during the fermentation process. Before ensiling, the mixtures were treated with compound lactic acid bacteria (LAB) inoculants followed by 3, 7, 14, 30, and 60 days of fermentation. The results revealed that the inoculated homofermentative species Lactobacillus plantarum and Lactobacillus farciminis dominated the early phase of fermentation, and these shifted to the heterofermentative species Lactobacillus buchneri and Lactobacillus hilgardii in the later phase of fermentation. As a result, the pH of the mixed silages decreased significantly, accompanied by the growth of acid-producing microorganisms, especially *L. buchneri* and *L. hilgardii*, which actively influenced the bacterial community structure and metabolic pathways. Moreover, the contents of lactic acid, acetic acid, 1,2-propanediol, and water-soluble carbohydrates increased, while the contents of ammonia-N and fiber were decreased, with increasing ratios of sweet sorghum in the mixed silage. Overall, coensiling *Sesbania cannabina* with >30% sweet sorghum is feasible to attain high-quality silage, and the relay action between homofermentative and heterofermentative LAB species could enhance fermentation quality and conserve the nutrients of the mixed silage.

**IMPORTANCE** The coensiling of *Sesbania cannabina* and sweet sorghum is of great practical importance in order to alleviate the protein-rich forage deficiency in saline-alkaline regions. Furthermore, understanding the microbial community’s dynamic changes, interactions, and metabolic pathways during ensiling will provide the theoretical basis to effectively regulate silage fermentation. Here, we established that coensiling *Sesbania cannabina* with >30% sweet sorghum was effective at ensuring better fermentation quality and preservation of nutrients. Moreover, the different fermentation types of LAB strains played a relay role during the fermentation process. The homofermentative species *L. plantarum* and *L. farciminis* dominated in the early phase of fermentation, while the heterofermentative species *L. buchneri* and *L. hilgardii* dominated in the later phase of fermentation. Their relay action in *Sesbania cannabina*-sweet sorghum mixed silage may help to improve fermentation quality and nutrient preservation.

## INTRODUCTION

With the rapid economic development of China, the demand for livestock products is gradually increasing. The lack of protein-rich forage in saline-alkaline regions is the primary constraint on animal husbandry development. This scarcity is usually caused by soil stress affecting typical plant nutrient uptake (1). Therefore, it is imperative to exploit high-protein forage in saline-alkaline regions to alleviate the deficiency in protein-rich forage. Sesbania cannabina is an annual herbaceous legume distinguished by its higher protein content (up to about 25% dry matter [DM]), higher DM yield (45 t ha^−1^ per year), and resistance to abiotic stresses such as salt and alkali stresses (2–5). Therefore, it is practical to use *Sesbania cannabina* as roughage to meet the increasing demand for high-protein forage in saline-alkaline regions.

*Sesbania cannabina* has a seasonal harvest with high biomass accumulation, which makes it feasible for making silage (1). Moreover, ensiling could preserve the nutritional components, improve the palatability, and prolong the storage time of forage (6). However, the application of *Sesbania cannabina* in the silage industry is limited because of its low water-soluble carbohydrate (WSC) content (less than 5% DM) and high buffer capacity. On the other hand, coensiling is a common practice to improve fermentation quality and enhance the stability of the fermentation system (7, 8). Numerous studies have reported the benefits of coensiling of sweet sorghum [Sorghum dochna (Forssk.) Snowden] (rich in WSC) with high-protein forage in terms of quality silage production (1, 7, 9). WSC-rich forage could provide the substrate for acid-producing microbes, especially lactic acid (LA) bacteria (LAB), which contribute to the reduction of pH. Thus, protein-degrading bacteria were inhibited, converting the crude protein (CP) in protein-rich forage (7). Besides, sweet sorghum is also suitable for cultivation in saline-alkaline regions and could be harvested simultaneously with *Sesbania cannabina* (1). Therefore, coensiling of *Sesbania cannabina* with sweet sorghum is a realistic way to obtain high-quality silage in saline-alkaline regions.

LAB additives are widely used for quality silage production because of their abilities to enhance LA, decrease the pH, inhibit protein degradation, and reduce DM loss in an airtight environment (10, 11). Generally, homofermentative LAB strains are the major contributors to lactic acid fermentation due to their higher efficiency of sugar utilization (12). A previous study reported that the rapid growth of Lactobacillus plantarum at the initial ensiling stage is vital for later fermentation and final silage quality (13). Heterofermentative LAB strains are known for their antifungal ability to improve silage aerobic stability by producing acetic acid (AA) and 1,2-propanediol (1,2-PD) (14, 15). However, much is still unknown about the succession of the homofermentative and heterofermentative LAB species and their functional shifts during the coensiling of *Sesbania cannabina* and sweet sorghum, which is essential for the further regulation of fermentation.

Consequently, the current study aimed to investigate the fermentation characteristics; bacterial community succession, especially the inoculated LAB species; and dynamic changes in the predicted microbial functions in mixed silage of *Sesbania cannabina* and sweet sorghum.

## RESULTS

### Fermentation characteristics of mixed silage of *Sesbania cannabina* and sweet sorghum during the ensiling process.

**FIG 1:**
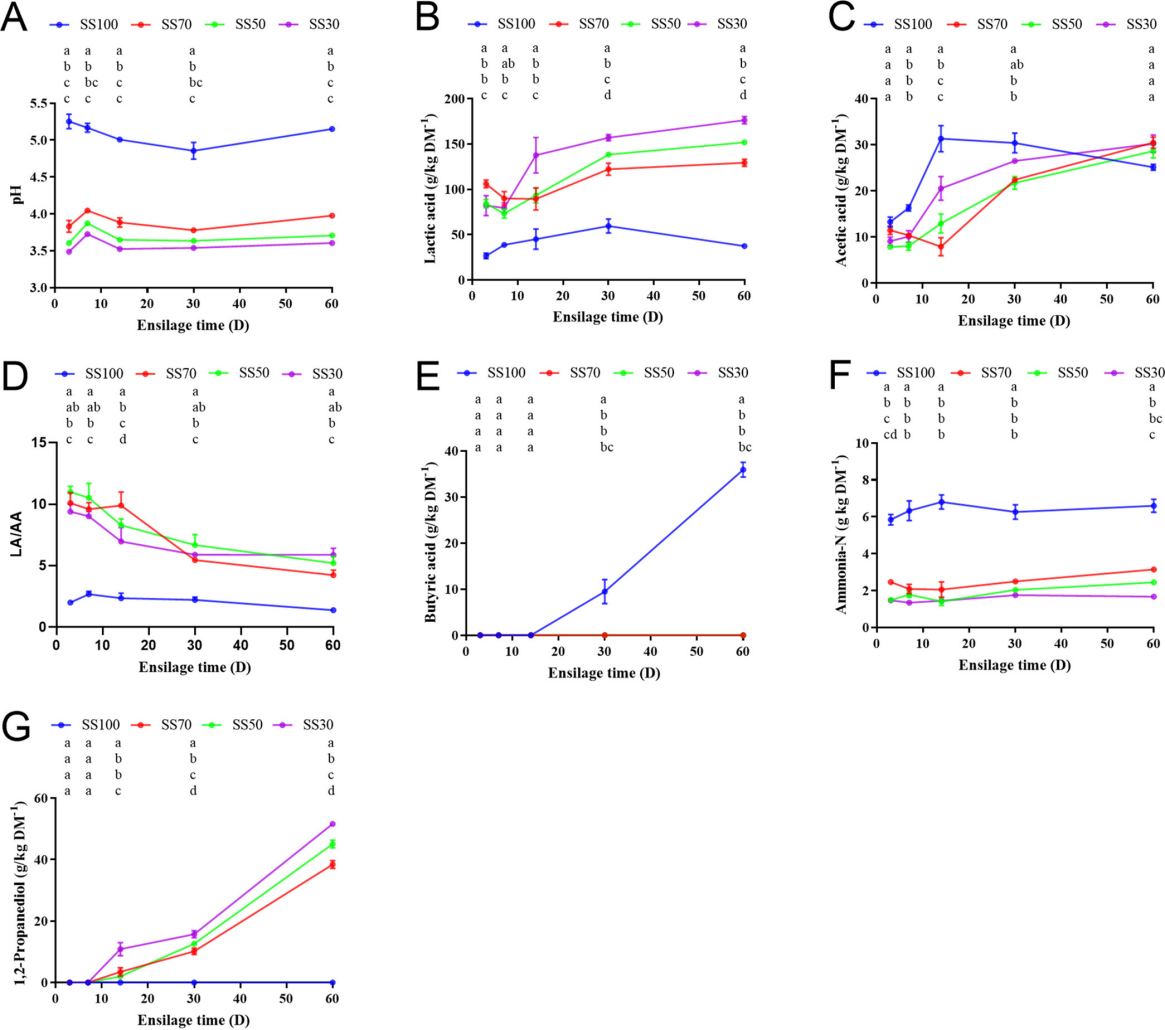
Dynamic changes in pH values (A) and contents of organic acids (B to E), ammonia-N (F), and 1,2-propanediol (G) during the ensiling process. LA, lactic acid; AA, acetic acid; SS100, 100% *Sesbania cannabina* without sweet sorghum; SS70, 70% *Sesbania cannabina* with 30% sweet sorghum; SS50, 50% *Sesbania cannabina* with 50% sweet sorghum; SS30, 30% *Sesbania cannabina* with 70% sweet sorghum.

The pH values of the mixed silages (SS70 [70% *Sesbania cannabina* with 30% sweet sorghum], SS50, and SS30 groups) were significantly (*P < *0.001) lower than that of the *Sesbania cannabina* silage (SS100 [100% *Sesbania cannabina* without sweet sorghum] group) throughout the ensiling period (Fig. 1). More specifically, rapid acidification was seen in the mixed silages at the initial phase of fermentation, and they maintained a relatively stable pH value (<4.2) until the last phase of fermentation. However, the pH value of the SS100 group remained above 5.1 at the end of ensiling. The mixed silages had higher LA contents (*P *< 0.001) and lower ammonia-N (AN) contents (*P *< 0.001) than those of the SS100 group. The SS100 group showed a higher AA content (*P *< 0.001) than those for the other treatments before 30 days of ensiling; however, there was no significant difference in AA contents among treatments at the final phase of fermentation. Butyric acid (BA) was found only in the SS100 group after day 30 of ensiling and reached up to 35.90 g/kg DM at the final phase of fermentation. 1,2-PD was detected in all groups except SS100, and it began to accumulate after 7 days of ensiling and kept increasing continuously throughout the fermentation process. Overall, the mixed-silage groups represented a better fermentation quality than the *Sesbania cannabina* silage. Moreover, the pH value and the AN content of mixed silage decreased with increasing ratios of sweet sorghum in mixed silage, leading to increased contents of LA and 1,2-PD.

### Chemical composition of mixed silage of *Sesbania cannabina* and sweet sorghum after 60 days of ensiling.

The interactions of ratios with ensiling were significant (*P *< 0.05) for the DM, CP, neutral detergent fiber (NDF), acid detergent fiber (ADF), acid detergent lignin (ADL), and WSC contents after 60 days of ensiling (Table 1). Compared to the fresh material, the contents of DM (+4.14%), ADF (+3.97%), and ADL (+27.22%) were significantly (*P *< 0.05) increased while the contents of CP (−8.18%), NDF (−2.43%), and WSC (−72.32%) were substantially (*P *< 0.05) decreased in the SS100 group after day 60 of ensiling. The DM content of mixed silages was marginally affected by the sweet sorghum ratios. The CP, ADF, and ADL contents increased while the NDF and WSC contents decreased with increasing ratios of sweet sorghum in mixed silages. Overall, the SS70 group showed a higher CP content (16.62% DM) and a lower NDF content (54.07% DM) than the other treatments, highlighting that it is the best mixed ratio to preserve the chemical characteristics of silage.

**TABLE 1:** Chemical compositions of fresh material and silage samples^*a*^

Component	Treatment	Mean content (% DM) ± SD		*P* value			
		Before ensiling	After 60 days of ensiling	SEM	R	E	R × E
DM	SS100	22.95 ± 0.07 Bc	23.90 ± 0.27 Ad	0.05	<0.001	<0.001	<0.001
	SS70	23.30 ± 0.17 Bb	24.80 ± 0.11 Ab				
	SS50	23.43 ± 0.09 Ba	25.30 ± 0.28 Aa				
	SS30	23.23 ± 0.15 Bb	24.40 ± 0.10 Ab				

CP	SS100	18.10 ± 0.24 Aa	16.62 ± 0.47 Bb	0.07	<0.001	0.12	<0.001
	SS70	15.50 ± 0.28 Bb	16.92 ± 0.31 Aa				
	SS50	13.45 ± 0.08 Ac	13.70 ± 0.09 Ac				
	SS30	12.40 ± 0.11 Ad	12.58 ± 0.16 Ad				

NDF	SS100	59.20 ± 0.14 Aa	57.76 ± 0.98 Ba	0.25	0.15	<0.001	<0.001
	SS70	61.89 ± 1.85 Aa	54.07 ± 0.38 Bb				
	SS50	62.90 ± 1.96 Aa	54.82 ± 0.43 Bb				
	SS30	62.33 ± 0.05 Aa	55.25 ± 0.79 Bb				

ADF	SS100	47.30 ± 0.01 Ba	49.18 ± 1.16 Aa	0.20	<0.001	<0.05	<0.001
	SS70	46.02 ± 1.38 Aa	42.90 ± 0.30 Bb				
	SS50	44.60 ± 0.89 Ab	41.13 ± 0.31 Bbc				
	SS30	41.97 ± 0.02 Ab	39.11 ± 0.58 Bc				

ADL	SS100	11.57 ± 0.37 Ba	14.72 ± 0.53 Aa	0.13	<0.001	<0.001	<0.05
	SS70	9.66 ± 0.31 Bb	12.00 ± 0.31 Ab				
	SS50	9.25 ± 0.80 Bb	10.14 ± 0.49 Ac				
	SS30	7.36 ± 0.09 Bc	8.41 ± 0.11 Ad				

WSC	SS100	1.77 ± 0.03 Ad	0.49 ± 0.01 Bc	0.02	<0.001	<0.001	<0.001
	SS70	3.45 ± 0.14 Ac	1.00 ± 0.01 Bb				
	SS50	5.50 ± 0.02 Ab	1.01 ± 0.01 Bb				
	SS30	7.34 ± 0.24 Aa	1.39 ± 0.04 Ba				

a DM, dry matter; CP, crude protein; NDF, neutral detergent fiber; ADF, acid detergent fiber; WSC, water-soluble carbohydrate; R, ratio of sweet sorghum; E, ensiling time; R × E, interaction between the ratio of sweet sorghum and the ensiling time; SS100, 100% *Sesbania cannabina* without sweet sorghum; SS70, 70% *Sesbania cannabina* with 30% sweet sorghum; SS50, 50% *Sesbania cannabina* with 50% sweet sorghum; SS30, 30% *Sesbania cannabina* with 70% sweet sorghum. Different lowercase letters indicate a significant difference in the same column (*P *< 0.05), and different uppercase letters indicate a significant difference in the same row (*P *< 0.05).

### Dynamic changes in the microbial community during the ensiling process.

**FIG 2:**
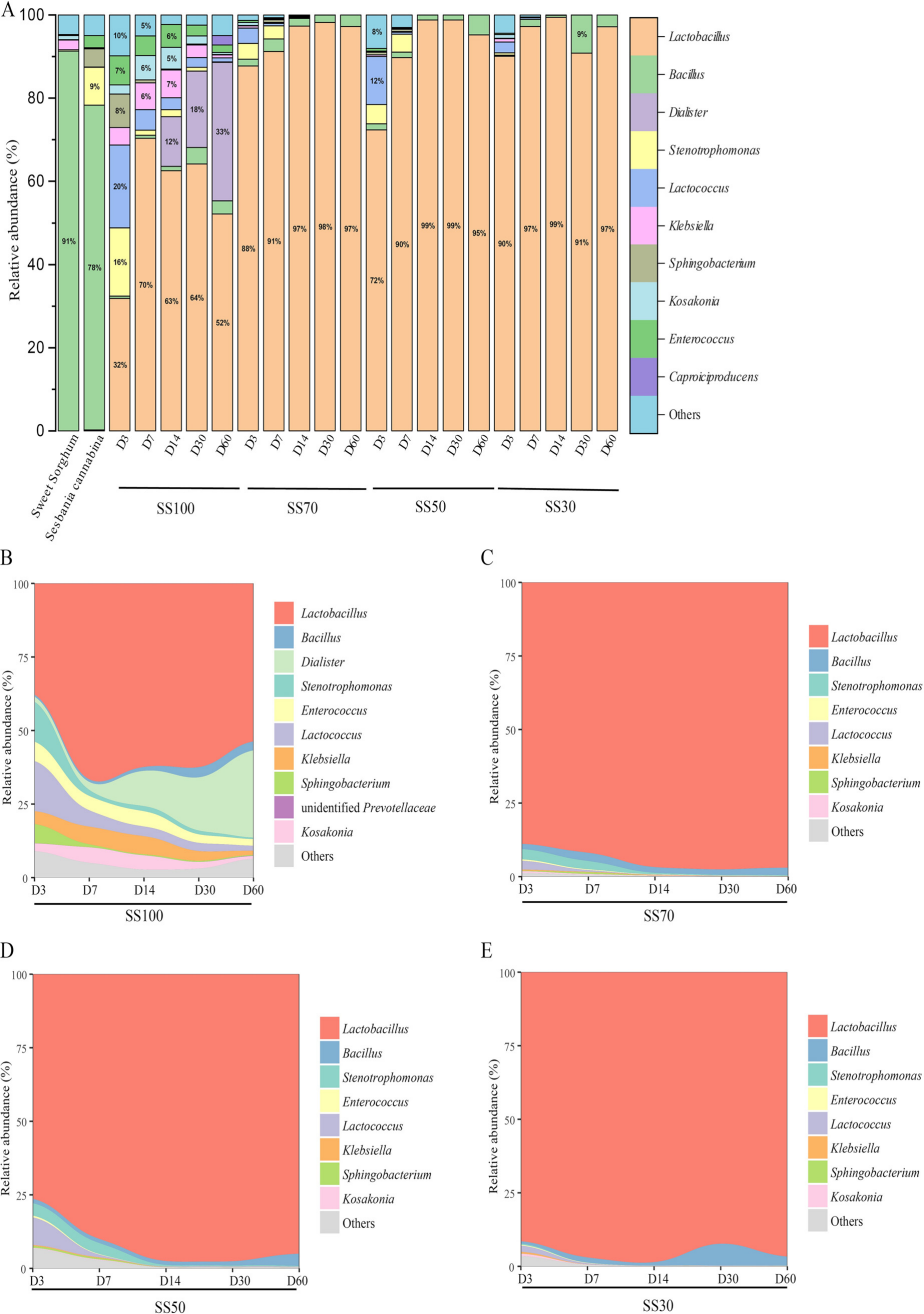
Bacterial community composition (A) and succession (B to E) at the genus level in silage during the ensiling process. SS100, 100% *Sesbania cannabina* without sweet sorghum; SS70, 70% *Sesbania cannabina* with 30% sweet sorghum; SS50, 50% *Sesbania cannabina* with 50% sweet sorghum; SS30, 30% *Sesbania cannabina* with 70% sweet sorghum.

The Chao1 and Shannon indices were gradually reduced in all treatments during the ensiling process (see Table S1 in the supplemental material). Meanwhile, lower Chao1 and Shannon indices were seen in mixed silages than in SS100 silage throughout the fermentation process. The bacterial community composition and succession at the genus level of the silages are shown in Fig. 2. *Bacillus* (78.05% in *Sesbania cannabina*; 91.37% in sweet sorghum) was the most predominant genus in the fresh materials, followed by the genera *Stenotrophomonas* and Klebsiella. Notably, the genus *Lactobacillus* was present at <1% in fresh materials, but it gradually became the most prevalent genus during the ensiling process. The relative abundance of *Lactobacillus* exceeded 95% in mixed silages compared to SS100 silage at the final phase of the ensiling process (Fig. 2A). According to the stream graph, the ratios of sweet sorghum had a considerable influence on the succession of bacterial communities in mixed silages.

**FIG 3:**
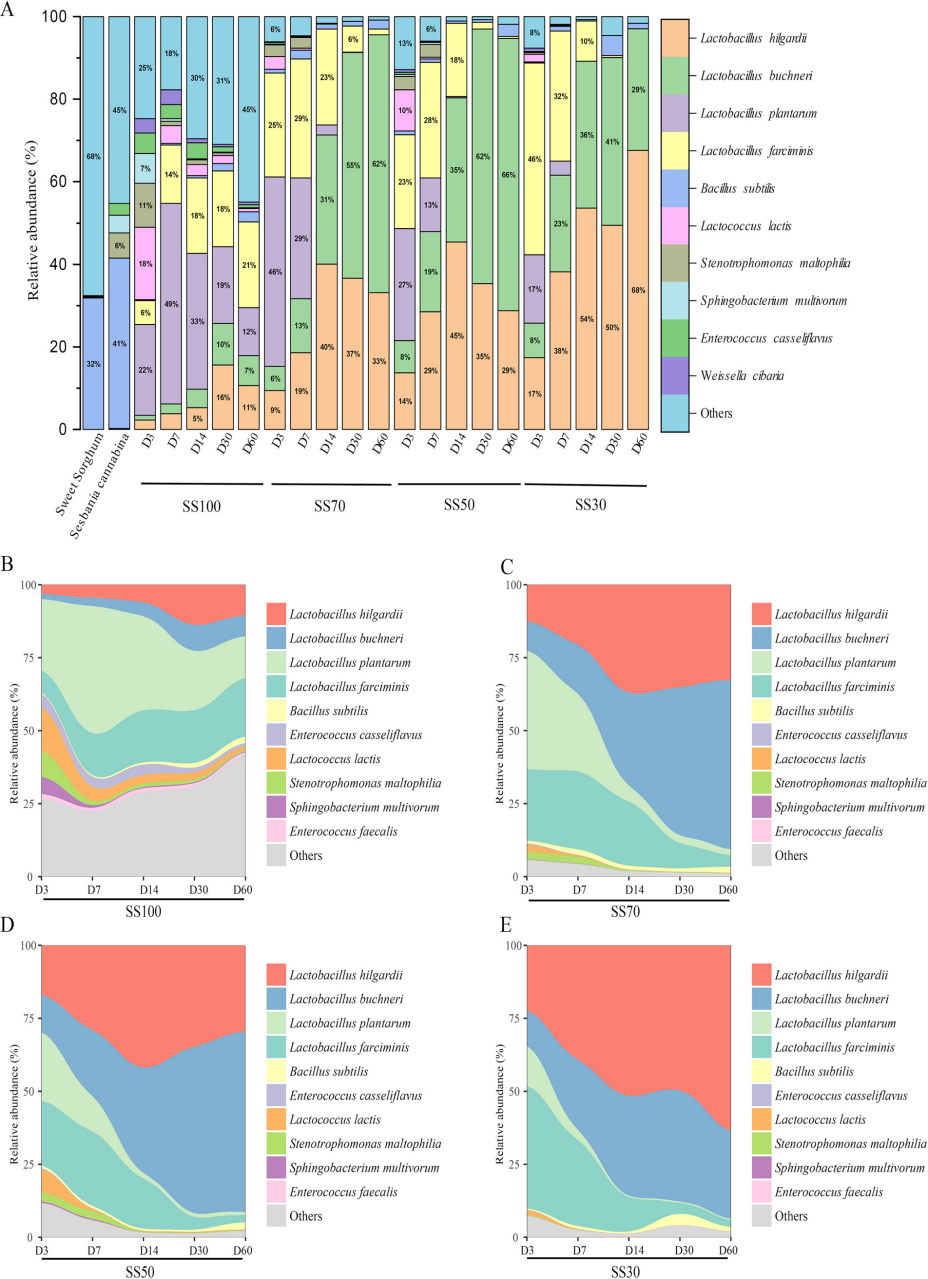
Bacterial community composition (A) and succession (B to E) at the species level in silage during the ensiling process. SS100, 100% *Sesbania cannabina* without sweet sorghum; SS70, 70% *Sesbania cannabina* with 30% sweet sorghum; SS50, 50% *Sesbania cannabina* with 50% sweet sorghum; SS30, 30% *Sesbania cannabina* with 70% sweet sorghum.

The dynamic changes in bacterial community composition and succession at the species level in silages are shown in Fig. 3. Bacillus subtilis was the prevalent bacterial species in the fresh materials, accounting for relative abundances of 41.29% in *Sesbania cannabina* and 31.83% in sweet sorghum (Fig. 3A). The relative abundance of Bacillus subtilis was decreased to nearly 1% with prolonged ensiling times, and similarly, the relative abundances of other undesirable microbes, like Stenotrophomonas maltophilia, Sphingobacterium multivorum, and Enterococcus casseliflavus, were also decreased. As expected, L. plantarum, Lactobacillus farciminis, Lactobacillus hilgardii, and Lactobacillus buchneri became the dominant species, with an average relative abundance of over 20% in all treatments after ensiling. In the SS100 group, the relative abundance of *L. plantarum* reached its highest level of 48.53% at day 7 of ensiling and then decreased to 11.54% at day 60 of ensiling. However, the relative abundance of *L. farciminis* kept increasing throughout the fermentation process and reached 20.83% at day 60 of ensiling. The relative abundances of *L. hilgardii* and *L. buchneri* kept increasing with prolonged ensiling times up to day 30 of ensiling and then decreased significantly to 10.62% and 7.29% at day 60 of ensiling, respectively (Fig. 3B). *L. plantarum* and *L. farciminis* were the most predominant bacterial species in the mixed silages at the early phase (before day 7) of ensiling. However, *L. hilgardii* and *L. buchneri* replaced them as the dominant species at the later phase (after day 7) of ensiling. Moreover, the relative abundance of *L. plantarum* reached its highest level earlier than *L. farciminis*, while the relative abundance of *L. buchneri* reached its highest level later than *L. hilgardii*. Overall, drastic dynamic changes in the bacterial community were seen in the SS100 silage during the ensiling process. However, the dynamic changes in the bacterial community tended to be flat after day 7 of ensiling in the mixed silages. In addition, the homofermentative LAB species dominated in the early phase of fermentation, while the heterofermentative LAB species dominated in the later phase of fermentation.

### Bacterial cooccurrence and cooccurrence network complexity and stability during the ensiling process.

**FIG 4:**
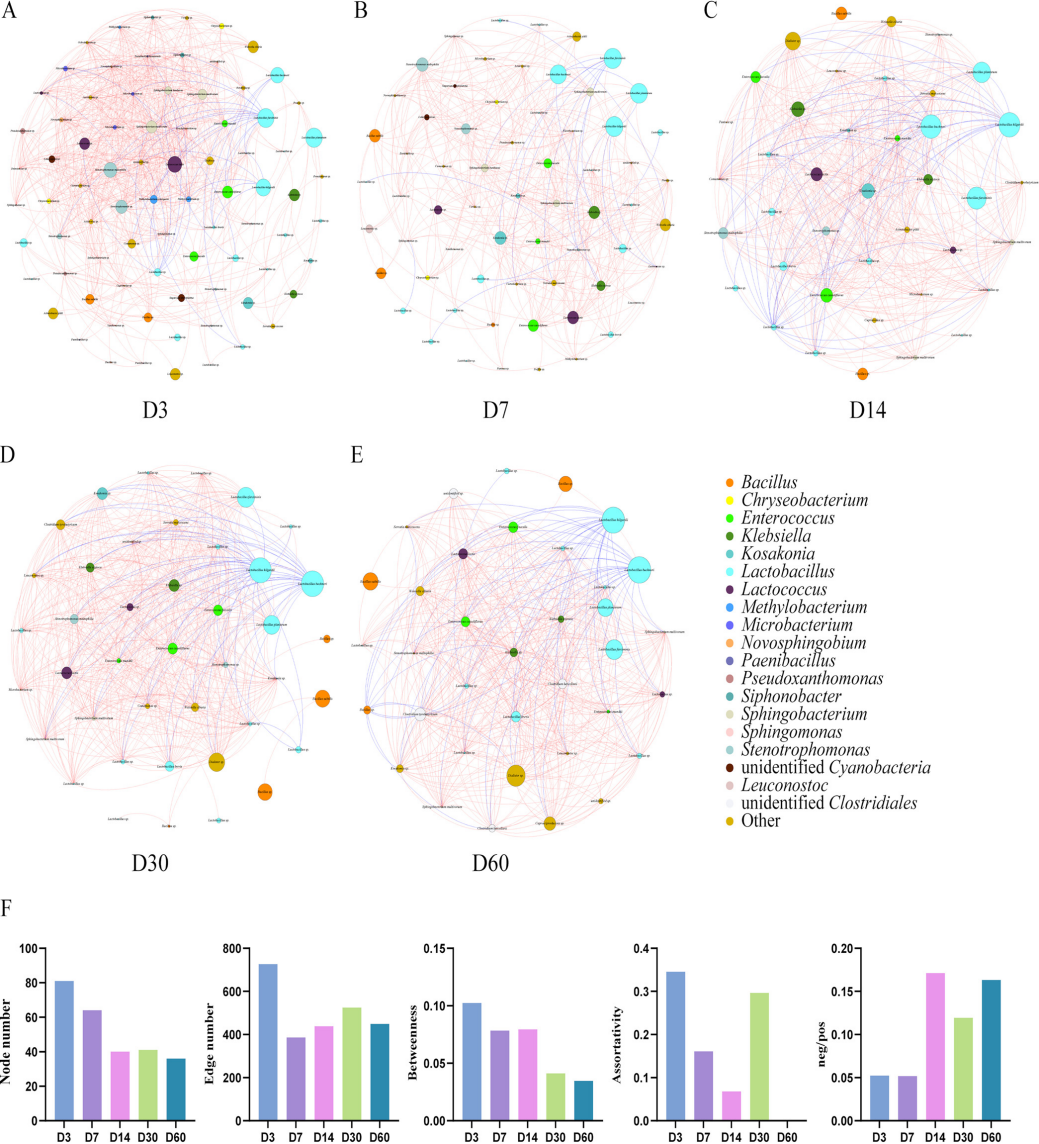
Cooccurrence patterns of the silage bacterial community during the ensiling process. (A to E) Cooccurrence patterns of bacterial communities at day 3 (D3), day 7, day 14, day 30, and day 60 of ensiling. (F) Numbers of nodes and edges, degrees of betweenness and assortativity, and negative/positive correlation (neg/pos) ratios of silage bacterial cooccurrence patterns.

The network of silage bacterial communities at each storage time demonstrated distinct occurrence patterns (Fig. 4). To assess bacterial network complexity, the numbers of nodes and edges and the degrees of betweenness and assortativity were used as network topological parameters. Moreover, negative/positive correlation ratios were used to evaluate bacterial network stability, with higher negative/positive correlation ratios representing greater network stability. After 3 days of fermentation, the bacterial network became simple, with fewer nodes and edges. However, bacterial network complexity changed irregularly along with storage time. Bacterial network stability was greater in the later phase (after day 7) of ensiling than in the early phase (before day 7).

### Predicted metabolic functions of the bacterial community during the ensiling process.

**FIG 5:**
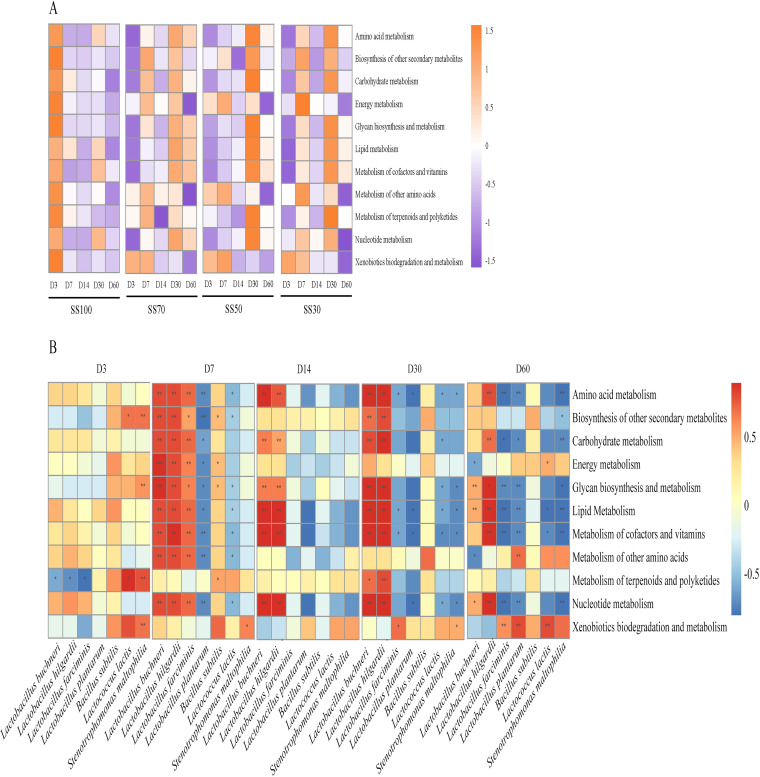
Predicted metabolic functional shifts during the ensiling process (A) and analysis of the association between bacterial species and metabolism pathways (B) in silage. SS100, 100% *Sesbania cannabina* without sweet sorghum; SS70, 70% *Sesbania cannabina* with 30% sweet sorghum; SS50, 50% *Sesbania cannabina* with 50% sweet sorghum; SS30, 30% *Sesbania cannabina* with 70% sweet sorghum. The correlations > 0.5 and < 0.5 were annotated significantly. *P* values are shown as *, *P* < 0.05; **, *P* < 0.01.

The sweet sorghum ratios significantly modulated the bacterial communities of mixed silage compared to the SS100 group, which contributed to striking differences in the predicted metabolic functions with prolonged ensiling times (Fig. 5A). In the SS100 group, all of the genes involved in metabolism were obviously upregulated at day 3 of ensiling, and most of them were then downregulated in the later ensiling phase. Metabolic functional gene upregulation in the mixed silages occurred mainly at day 7 and day 30 of ensiling. For example, energy metabolism and the metabolism of other amino acids were upregulated at day 7, while amino acid, carbohydrate, and lipid metabolisms were upregulated at day 30. In comparison, within the mixed silages, only the SS70 group had some slightly upregulated functional metabolic pathways at day 60 of ensiling, such as amino acid metabolism, lipid metabolism, glycan biosynthesis and metabolism, and the metabolism of cofactors and vitamins.

In order to examine the contribution of bacteria to the predicted metabolic pathways during the fermentation process, the correlation between the bacterial species (relative abundance of >1%) and the predicted metabolic functions was analyzed (Fig. 5B). At day 7 of ensiling, *L. buchneri*, *L. hilgardii*, and *L. farciminis* were positively correlated with most of the metabolic functions such as amino acid, carbohydrate, and energy metabolism. Furthermore, it was fascinating to determine that only *L. buchneri* and *L. hilgardii* continued to influence the metabolism pathways with prolonged ensiling times, highlighting that these species have the potential to affect the bacterial community’s metabolic pathways.

### Correlations among bacterial species, predicted metabolic functions, and fermentation quality of products.

**FIG 6:**
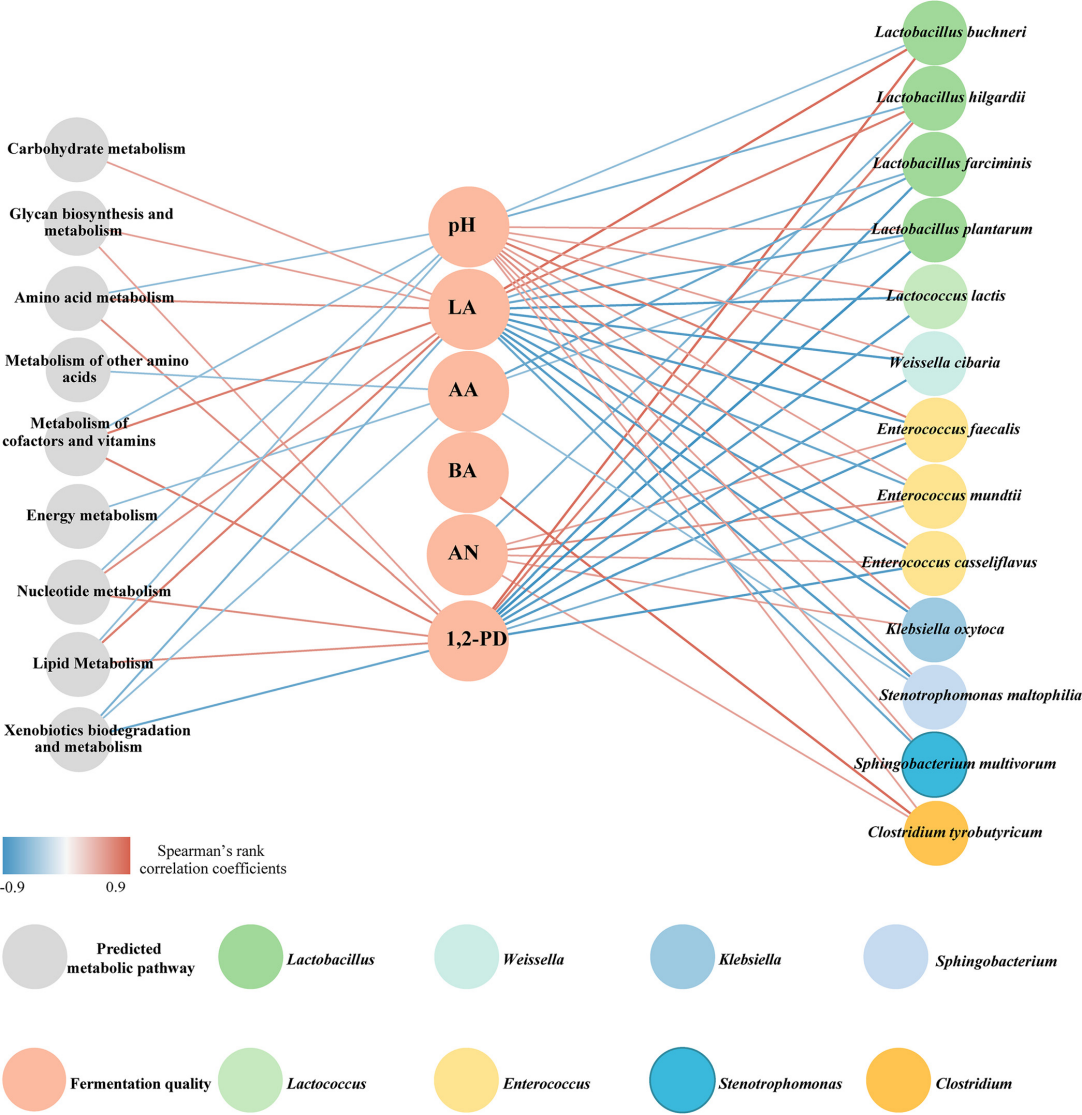
Correlation network plots for bacterial species, predicted metabolic functions, and fermentation quality. The predicted metabolic functions, fermentation quality, and bacterial species are presented as dots. The edge width is proportional to the strength of the association (as measured by the correlation); a red edge indicates a positive correlation, and a blue edge indicates a negative correlation. LA, lactic acid; AA, acetic acid; BA, butyric acid; AN, ammonia-N; 1,2-PD, 1,2-propanediol.

In order to further investigate the effects of bacterial species and predicted metabolic functions on silage fermentation quality, a correlation analysis among species, predicted metabolic functions, and fermentation characteristics was conducted (Fig. 6). pH was positively correlated with many species of *Enterococcus* (such as E. casseliflavus, E. faecalis, and E. mundtii) and Klebsiella oxytoca, while it was negatively correlated with the species *L. buchneri* and *L. hilgardii* (Table S2). The higher pH value and higher AN and BA contents are detrimental to fermentation quality. The species *E. casseliflavus*, E. faecalis, *E. mundtii*, and Klebsiella oxytoca displayed positive correlations with BA and AN, and Clostridium tyrobutyricum showed a positive correlation with BA (Table S3). Many predicted metabolism functions showed a trend toward negative correlations (*R* value of less than −0.5) with BA and AN, such as amino acid metabolism, carbohydrate metabolism, glycan biosynthesis and metabolism, lipid metabolism, the metabolism of cofactors and vitamins, and nucleotide metabolism (Table S3). Moreover, pH and LA were closely correlated with the metabolism of amino acids, lipids, nucleotides, cofactors, and vitamins, and a positive correlation was found between these predicted metabolism functions and LA, while a negative correlation was found with pH (Table S3).

## DISCUSSION

The silage quality of forage is driven mainly by microbial activity and is greatly dependent on the substrates available in fresh materials (6, 16). The pH of silage is considered a critical indicator for silage fermentation (6). In the present study, the pH of the mixed silages decreased significantly during the initial day 3 of ensiling, probably because of sweet sorghum ratios in such silages, which provided a sufficient substrate for LAB to initiate fermentation. However, the pH of mixed silages became stable with prolonged ensiling times and was below 4.2. It is widely acknowledged that a low pH value of 4.2 is considered a benchmark for well-preserved silage (7). In contrast, *Sesbania cannabina* silage had a higher pH value, which might be related to the lower LA content in this silage. The increase in the pH and the decrease in the LA concentration from days 3 to 7 in mixed silages could be attributed to the sudden decrease in the relative abundance of homofermentative LAB (*L. plantarum* and *L. farciminis*). A previous study reported that AA is the major end product of heterofermentative LAB (17). In the present study, AA production began to increase after day 7 of ensiling, accompanied by increasing relative abundances of the heterofermentative species *L. buchneri* and *L. hilgardii*, which was consistent with previous work (18). However, the pH value decreased again from days 7 to 14 in the mixed silage, which might be because of the increased relative abundances of four LAB species (*L. plantarum*, *L. farciminis*, *L. buchneri*, and *L. hilgardii*), and the low pH value in the mixed silage later may be due to the higher relative abundances (over 90%) of these four LAB species. BA is usually produced by undesirable bacteria that decompose CP, resulting in nutrient loss. In the present study, BA was detected only in the SS100 silage after day 30 of ensiling, which could be related to the presence of the species Clostridium tyrobutyricum in this silage. A previous study reported that the species *C. tyrobutyricum* could survive in silage with a pH higher than the benchmark value of 4.2 (19); therefore, the higher pH of *Sesbania cannabina* silage could not suppress its growth. The accumulation of AA and 1,2-PD increased with increasing abundances of heterofermentative LAB in the mixed silages, in agreement with data from previous studies (20–22). This could be attributed to the conversion of LA to AA and 1,2-PD by *L. hilgardii* and *L. buchneri* under acidic conditions (23, 24).

The chemical compositions of the silages were significantly affected after ensiling. The DM contents of the silages were little influenced by the fermentation process. It is worth noting that the CP content was obviously reduced in the *Sesbania cannabina* silage, which might be related to the extensive proteolysis resulting from clostridial fermentation or plant enzyme activity (6). The level of AN is an indicator of CP degradation in silage. The AN content of the mixed silages was significantly lower than that of the SS100 silage, indicating that undesirable proteolytic bacteria were inhibited effectively in the mixed silages. The CP content was better preserved in the mixed silages, which could be attributed to the sharp decline in the pH in such silages that inhibited the growth of undesirable microorganisms. The ADF and NDF contents decreased while the ADL content increased after ensiling in all groups. This could be exemplified by the more digestible cell wall fractions hydrolyzed by enzymes and acids during silage fermentation (25). The contents of WSC decreased in all treatments after ensiling, indicating that it was utilized and broken down into organic acids by LAB during silage fermentation. The trend of decreasing WSC contents for all of the treatments was consistent with the pH value.

Silage fermentation is mainly a bacterium-driven process and is largely dependent on the composition and structure of the bacterial community. Bacillus subtilis was the dominant bacterium in the fresh materials of *Sesbania cannabina* and sweet sorghum. Generally, *Bacillus* bacteria compete with LAB for sugars, and their presence in silage is considered undesirable (26). However, the growth of *Bacillus* was inhibited significantly after ensiling, which might be related to the reduction in the pH in all treatments. The succession of bacteria is a dynamic process that varies in different phases of fermentation. It has been reported that cocci (such as *Lactococcus*, *Leuconostoc*, *Pediococcus*, and *Enterococcus* spp.) are the starters of LA-type fermentation during the early phase of ensiling and are usually replaced by rod-shaped lactic acid bacteria (such as *Lactobacillus*) at the later phase of ensiling (27, 28). In this study, the relative abundance of Lactococcus lactis was increased during the initial fermentation stage, but it was lower than those of the inoculated *L. plantarum* and *L. farciminis*. This could be ascribed to differences in competitiveness between the exogenous LAB and native microorganisms. The species *L. plantarum* and *L. farciminis* grew significantly in the SS100 silage, although the fermentation substrate was deficient for this group. This might be because of the anaerobic conditions that facilitated the growth of LAB strains, which could produce LA to enhance their competitiveness by inhibiting other background bacteria (29). It was fascinating to observe higher relative abundances of *L. plantarum* with higher ratios of *Sesbania cannabina* in mixed silages but a higher relative abundance of *L. farciminis* with higher ratios of sweet sorghum in mixed silages during the early fermentation phase. This might be related to the differences in substrate environments, as the protein-rich environment (more *Sesbania cannabina*) was feasible for the growth of *L. plantarum*, and the WSC-rich environment (more sweet sorghum) was suitable for the growth of *L. farciminis.* However, the relative abundances of the homofermentative species *L. plantarum* and *L. farciminis* were decreased in the mixed silage with prolonged ensiling times, which might be due to rapid acidification that suppressed their growth (30). On the contrary, the relative abundances of the heterofermentative species *L. hilgardii* and *L. buchneri* increased after day 7 of ensiling, which agrees with previous work (31). Although a previous study explored the dynamic changes in the microbiota in whole plant corn silage inoculated with *L. buchneri* and *L. hilgardii*, it did not explain the order of succession of *L. buchneri* and *L. hilgardii* (32). In the present study, the relative abundances of *L. buchneri* and *L. hilgardii* tended to increase with increasing ensiling times, and the relative abundance of *L. hilgardii* reached its highest level earlier than *L. buchneri.* At the end of fermentation, *L. buchneri* and *L. hilgardii* were the dominant species in mixed silages, but these species failed to dominate in the SS100 silage. Moreover, a lower relative abundance of LAB was observed in the SS100 silage than in the other treatments, resulting in slower acidification and weaker LA-type fermentation due to the sugar-deficient environment.

Environmental bacterial communities play a vital role in ecosystem function, and the interactions among microorganisms are complex in the silage fermentation ecosystem (33). High numbers of nodes and edges represent greater network complexity (34). The current study demonstrated that the microbial network was more complex at the initial phase of fermentation (at day 3), which might be due to the presence of undesirable microorganisms. Bai et al. (18) found higher negative/positive correlation ratios in corn silage, highlighting that the bacterial network structure was more stable in corn silage. In the present study, a higher negative/positive correlation ratio was found in the mixed silages after day 3, suggesting that bacterial network stability was greater in the later phase of fermentation than in the initial phase. This bacterial network stability could be attributed to *L. buchneri* and *L. hilgardii* domination in the later phase of ensiling, as their activities are negatively correlated with most of the bacteria. Similarly, a previous study reported that the bacterial community network in alfalfa silage treated with LAB was more stable after day 7 of fermentation (35).

Ensiling is a complex process involving various microbial metabolic pathways, and the end products are essential parameters of silage fermentation quality. The prediction of functional shifts during the ensiling process reflects the effects of the bacterial community on the dynamic changes in metabolic pathways (36). Almost all of the metabolic pathways were downregulated in *Sesbania cannabina* silage after day 7, indicating a decrease in microbial activity. On the contrary, metabolic pathways associated with fermentation (metabolism of amino acids, carbohydrates, and lipids) remained upregulated in the mixed silage at the later phase (day 30) of fermentation. Metabolisms of amino acids, carbohydrates, cofactors, and vitamins were positively correlated with LA, the main product in high-quality fermented silage. These upregulated metabolic pathways are indicators of continuous bacterial activity, which breaks down polysaccharides within the plants into monosaccharides and LA (35). This may provide valuable clues for targeting specific metabolic pathways in order to regulate the fermentation quality of silages. LA was positively correlated with *L. buchneri* and *L. hilgardii*, while it was negatively correlated with *L. plantarum* and *L. farciminis*. Similarly, Xu et al. (36) also reported a negative correlation between *L. plantarum* negativity and LA in corn silage treated with or without *L. plantarum*. This may account for the decreased relative abundances of *L. plantarum* and *L. farciminis* in all treatments along with the ensiling process. LA was also negatively correlated with many species of *Enterococcus*, consistent with previous reports by Yang et al. (13) and Xia et al. (1). Silages with the production of BA and AN are regarded as poor-quality silages with unpleasant palatability and low nutritional value. According to the correlation analysis, the undesirable microorganism that contributed to BA and AN production was *C. tyrobutyricum*, while those that promoted AN production were *E. casseliflavus*, E. faecalis, and *E. mundtii*.

### Conclusion.

The coensiling of *Sesbania cannabina* and >30% sweet sorghum confirmed the benefits of enhancing silage quality by providing sufficient WSC, enhancing the relative abundance of inoculated LAB strains, and restricting the growth of unwanted microbes such as *Clostridium* and *Enterococcus* in mixed silage. The homofermentative species *L. plantarum* and *L. farciminis* were the dominant species in the early phase of fermentation and were then replaced by the heterofermentative species *L. buchneri* and *L. hilgardii* in the later phase of fermentation. Moreover, *L. buchneri* and *L. hilgardii* had obvious effects by manipulating the bacterial community and predicted metabolic functions. Taken together, these results suggested that the relay action of the different fermentation types of LAB strains may help to improve fermentation quality and nutrient preservation. Overall, this study explained the succession mechanism of exogenous LAB inoculants in mixed silage, which could facilitate the production of *Sesbania cannabina* silage and alleviate the demands for high-protein forage in saline-alkaline regions.

## MATERIALS AND METHODS

### Forage harvest and silage preparation.

Fresh materials were collected from the experimental field of the Yellow River Delta Modern Agricultural Technology Innovation Center located in Guangrao County, Dongying City, Shandong Province, China (37°67′N, 118°90′E), on 9 September 2020. *Sesbania cannabina* (var. *Lujing 5*) was harvested at the pod-bearing stage, while sweet sorghum (Sorghum dochna var. *Ketian 14*) was harvested at the milky ripe stage. The DM content of *Sesbania cannabina* was 29.7% fresh matter (FM), and its WSC, CP, NDF, ADF, and ADL contents were 1.77, 18.59, 52.68, 42.09, and 9.33% DM, respectively. The DM content of sweet sorghum was 25.1% FM, and its WSC, CP, NDF, ADF, and ADL contents were 23.54, 3.00, 49.67, 31.02, and 3.34% DM, respectively. The fresh raw materials were chopped into 2- to 3-cm pieces using a crop chopper (ZS-2; Zhongsheng Agricultural Machinery Company, Tangshan, China). After chopping, *Sesbania cannabina* and sweet sorghum were homogeneously mixed with compound LAB inoculants at four ratios: 100% *Sesbania cannabina* without sweet sorghum (SS100), 70% *Sesbania cannabina* with 30% sweet sorghum (SS70), 50% *Sesbania cannabina* with 50% sweet sorghum (SS50), and 30% *Sesbania cannabina* with 70% sweet sorghum (SS30). The compound LAB inoculants included *L. plantarum* B90, *L. farciminis* GMX4, *L. buchneri* NX205, and *L. hilgardii* 60TS-2, which were prepared according to methods described previously by Xia et al. (1). The mixed raw materials (500 g for each replicate) were packed manually into 32-cm by 45-cm polyethylene bags and tightly vacuum sealed. A total of 60 bags (4 ratios × 5 ensiling days × 3 replicates) were conserved at room temperature. The silage samples were obtained at days 3, 7, 14, 30, and 60 to evaluate the fermentation quality and microbial community. Only fresh and day 60 fermented silage samples were used to analyze the chemical composition.

### Fermentation quality and chemical composition analysis.

The 10-g silage samples at each storage time were blended with 90 mL sterilized water for 30 min. Next, the solution was passed through a filter (0.22 μm) to obtain the resulting filtrate to analyze the pH, organic acids, 1,2-PD, and AN (14). A glass electrode pH meter (Hanna, Italy) was used to determine the pH of the silages. The levels of organic acids were determined by high-performance liquid chromatography (HPLC) (ICSep Coregel-87H column, 210-nm UV detector, mobile stage of 0.005 M H_2_SO_4_ at 0.6 mL/min, and temperature of 55°C) (1200; Agilent, USA) (1). The concentrations of AN were measured according to methods described previously by Broderick and Kang (37). The silage samples were dried at 65°C for 48 h in a forced-air oven to estimate the DM content. Next, the dried samples were ground into particles with a diameter of 1.0 mm to analyze the contents of WSC, CP, NDF, ADF, and ADL according to the methods of the Association of Official Analytical Chemists (AOAC) (38).

### Sequencing and analysis of the bacterial community.

The 10-g silage samples were blended with 40 mL of a sterile phosphate-buffered saline (PBS) solution and then shaken at 120 rpm for 30 min at 4°C. The supernatant was filtered through four layers of gauze. Subsequently, the filtered supernatant was centrifuged at 12,000 rpm for 5 min at 4°C. After the above-described pretreatments, the bacterial precipitate was collected to extract the total genomic DNA using a DNA isolation kit (catalog number 18815ES50; Yeasen, Shanghai, China) (14, 39). The quantity and quality of the extracted DNA were measured using the NanoDrop ND-2000 spectrophotometer (Thermo Fisher Scientific, Waltham, MA, USA) and agarose gel electrophoresis, respectively. PCR amplification, amplicon purification, and quantification were performed according to methods described previously by Xia et al. (1). The PCR products were sequenced on a PacBio Sequel platform (Pacific Biosciences, Menlo Park, CA, USA) according to the standard protocols of Novogene (Beijing, China). Raw barcode data were obtained using lima software (v1.7.0) and revised by circular consensus sequencing and length filtering. The chimeras were removed by using UCHIME (v7.0.1090). The clean data were classified into operational taxonomic units (OTUs) based on a 97% threshold identity using Uparse software (v7.0.1001) (40). Subsequently, representative sequences were compared and annotated using the small-subunit (SSU) rRNA database (41). Alpha and beta diversity values were obtained using QIIME (version 1.9.1) based on the output-normalized data. The stream graph used to show bacterial community successions was calculated according to methods reported previously by Bai et al. (18). The cooccurrence networks were constructed by using the igraph and psych libraries in R and visualized by using Gephi (https://gephi.org) (42). The bacterial species with more than 5 OTUs were analyses.

### Predicted metabolic function analysis.

Bacterial function was predicted from the Kyoto Encyclopedia of Genes and Genomes (KEGG) database using Phylogenetic Investigation of Communities by Reconstruction of Unobserved States 2 (PICRUSt2), which predicts the functional abundance of samples based on the abundance of marker gene sequences in the sample (43). Heat map analysis was performed by using the pheatmap libraries in R.

### Bacterial species, predicted metabolic function, and fermentation quality correlation analyses.

Spearman’s correlation analysis between bacterial species, predicted metabolic functions, and fermentation quality was performed, and the network diagram was constructed by using Cytoscape (v3.7.1). To make the plot clear, only some of the data were screened and displayed. In brief, the top 15 bacterial species with the highest relative abundances were chosen, and only the variables with absolute *R* values of ≥0.5 and *P* values of <0.05 were shown.

### Statistical analysis.

The data are displayed as the means ± standard deviations (SD). The fermentation quality data at each ensiling time point were analyzed by one-way analysis of variance (ANOVA), and chemical composition data were analyzed by two-way analysis of variance. All of the statistical analyses were performed using SPSS 20 and GraphPad Prism 8.0. The means were compared for significance by Duncan’s multiple-range method, and significance was declared at a *P* value of <0.05.

### Data availability.

Raw sequencing files and associated metadata have been deposited in the NCBI Sequence Read Archive (accession number PRJNA839345).
